# A qualitative descriptive study on the alignment of care goals between older persons with multi-morbidities, their family physicians and informal caregivers

**DOI:** 10.1186/1471-2296-14-133

**Published:** 2013-09-08

**Authors:** Kerry Kuluski, Ashlinder Gill, Gayathri Naganathan, Ross Upshur, R Liisa Jaakkimainen, Walter P Wodchis

**Affiliations:** 1 Bridgepoint Collaboratory for Research and Innovation, Bridgepoint Active Healthcare, 14 St. Matthews Road, Toronto, ON M4M 2B5, Canada; 2 Institute of Health Policy, Management and Evaluation, University of Toronto, 155 College Street, Suite 425, Toronto, ON M5T 3M6, Canada; 3 Department of Family and Community Medicine, University of Toronto, 500 University Avenue, 5th Floor, Toronto, ON M5G 1V7, Canada; 4 Sunnybrook Academic Family Health Team, Sunnybrook Health Sciences Centre, 2075 Bayview Avenue, Toronto, ON M4N 3M5, Canada; 5 Institute for Clinical Evaluative Sciences, G1 06, 2075 Bayview Avenue, Toronto, ON M4N 3M5, Canada; 6 Toronto Rehabilitation Institute, 550 University Ave, Toronto, ON M5G 2A2, Canada

**Keywords:** Family practice, Multi-morbidity, Goals

## Abstract

**Background:**

Goal setting is a recommended approach in clinical care that can help individuals with multi-morbidities and their caregivers manage chronic conditions. In this paper, the types of goals that were important for older persons with multi-morbidities were explored from the perspectives of patients, their caregivers and physicians. Comparisons of goals were made across each patient, caregiver and physician triad to determine alignment.

**Methods:**

The study was a qualitative descriptive study facilitated through semi-structured one-on-one interviews. The study took place between May and October 2012 at a Family Health Team located in Ontario, Canada. The sample included 28 family medicine patients, their informal caregivers and family physicians. Socio-demographic data were analyzed via descriptive statistics in SPSS Version 17. Open ended questions pertaining to patient goals of care were analyzed thematically using NVivo9. Themes were derived on patient care goals for each of the participant groups (patients, caregivers and family physicians). Following this, alignment of goals across each of the triads was examined. Goal alignment was defined as concurrence on at least one goal by all three parties in a particular triad (i.e., patient, caregiver and family physician).

**Results:**

Just over half of the patients were male (56%); they had an average age of 82.3 years and 4.61 health conditions. Most of the caregivers were female (82%); and 61% were a spouse of the care recipient. At the aggregate level, common goals expressed among patients, caregivers and family physicians were the maintenance of functional independence of patients and the management of their symptoms or functional challenges. Despite these common goals at the aggregate level, little alignment of goals was found when looking across patient-caregiver and physician triads. Lack of alignment tended to occur when patients had unstable or declining functional or cognitive health; when safety threats were noted; and when enhanced care services were required.

**Conclusions:**

The data suggest that goal divergence tends to occur when patients are less medically stable. While goal divergence may be expected due to the different roles and responsibilities of each of the players involved, these perspectives should be illuminated when building care plans. Further research is required to observe the extent to which goal setting occurs in family practice as well as how it can be embedded as a standard of practice.

## Background

Over the last century, life expectancy has increased by up to forty years [1] with a marked increase in persons over the age of 65 [2]. The inherent trade-off to living longer is the greater likelihood of aging with one or more chronic illnesses [2,3]. Health care systems, with their acute, episodic orientation are limited in their capacity to provide care for patients with ongoing and fluctuating chronic care needs [3,4].

Widely recognized is a need to redesign or adapt the health care system to align with the changing needs of patients. Primary health care, a setting where much chronic disease management takes place, has been a focal point for reform across industrialized healthcare systems; showing potential in improving patient health outcomes [5] and alleviating strain on hospitals and emergency departments [6]. Key to the success of primary care is addressing the unique needs of each patient and providing them, and their family caregivers with the tools to manage their illnesses. A key challenge is that providers *themselve*s may lack the tools to manage patients, particularly if they have multi-morbidities [7]. Multi-morbidity is increasingly becoming the norm in older adult populations and is estimated to comprise up to 98% of the over 65 population seen in primary care practice [8]. Clinical practice guidelines which are primarily designed for single diseases have limited applicability for persons with multi-morbidity [7,9,10]. Physicians may be forced to make decisions that involve prioritization or trade-offs, warranting a discussion with the patient on what is important to them and what they would like to achieve in terms of their health (i.e. goal setting). Understanding patient’s goals of care can potentially aid in the successful management of their diseases at home [11] and when integrated into care plans, can improve their quality of life [12].

Goal setting is not necessarily a formal part of primary care practice. For example, a Canadian survey of the experiences of primary health care drawn from a nationally representative sample of 11,582 primary care patients with at least one chronic condition found that less than half of patients (48%) talked to their health care provider (at least some of the time) about their treatment goals [13]. International data involving goal setting between physicians and adults with chronic illness has noted similar trends [11]. Although rarely studied, some research has started to elucidate what patient goals may look like. For example, a study conducted in the US demonstrated that when older adults with multiple morbidities were asked about their care goals, 76% identified autonomy as the most important goal (to pain management, symptom relief, and staying alive) [14].

Failure to share goals raises a risk that physicians may focus on aspects of care and treatments that are not desired by the patient and/or family member [15]. Conversely, the patient and family may focus on things that the physician does not deem feasible [16]. Alignment in goals between patients, their caregivers and family physicians has rarely been studied [16], and seldom includes the perspective of the patient. Research by Heisler et al. [17] demonstrated that goal alignment can be associated with higher patient self-efficacy related to self-care. There are no published studies on goal alignment to our knowledge that involve older patients with multi-morbidities, their caregivers and family physicians. Given the increasing number of older persons and their caregivers who are aging in the community with multiple health challenges, exploring this topic is important in order to optimize self-management support and health outcomes.

The purpose of this study was to examine patient goals of care from the perspectives of older persons with multi-morbidities, their family physicians and informal caregivers (i.e., family member or friend who provides ongoing support) and then examine the extent of alignment between these three perspectives. The two research questions addressed are: 1) “*What are patient goals of care from the perspectives of older adults with multi*-*morbidities*, *their caregivers and family physicians*? 2) “*Do patient goals of care align among patient*-*caregiver and family physician triads*?” Goal alignment was defined as concurrence on at least one goal by all three parties in a particular triad (i.e., patient, caregiver and family physician).

## Methods

### Study design

The study design was qualitative description facilitated through semi-structured one-on-one interviews. Qualitative description is an established methodology which draws out informational content from the data and organizes it thematically in a way that is suitable for the research audience [18]. The interview guide was adapted from a previous study conducted by the lead author (KK) which elicited the perceived needs, and experiences from 116 hospitalized patients with multi-morbidity [19,20]. The interview guide was designed using a bio-psychosocial framework [21] and consisted of closed and open ended questions on physical functioning, disease prevalence and illness severity; social connectivity; mental health and overall experience of care [19,20]. The interview guide was pilot tested in this initial study and adapted until deemed feasible for use with a complex patient population. For the study reported in this paper, the researchers added two questions on patient goals of care and created two shorter interview guides for the physician and the caregiver with the same question on goals of care for the patient. Although the interview guide had other components this particular paper provides an in-depth analysis of the results of the question pertaining to goals of care. Other data generated from the interviews (e.g., health services use and care frustrations) will be reported in separate papers.

### Setting and participants

The study took place at a Family Health Team (a type of primary care model) in an urban community in Ontario, Canada between May and October 2012. A purposive sampling technique was used to select participants with the following characteristics: 65 years or older; ability to communicate in English; two or more chronic diagnoses; ability to give informed consent; an informal caregiver who agreed to participate in an interview. Two methods of patient recruitment were adopted: Family physicians first identified eligible patients from their patient rosters. The names of these patients were then cross-referenced with the patient-scheduling system to track when patients were visiting the Family Health Team. When an eligible patient had an appointment, the family physician first introduced the research study, and then asked whether the patient would like additional information from a Research Associate. If the patient said yes, the Research Associate provided additional details and acquired consent if the patient was amenable. This method of patient recruitment was quite slow so the two administrative assistants of the Family Health Team were later involved. The administrative assistants manage patient appointment scheduling, and other patient-related tasks. If the family physician identified an eligible patient who was not being seen in the Family Health Team within the next month, the administrative assistants phoned the patient at home, explained the research study and asked whether they would like to participate and be contacted by a Research Associate. If the patient said yes, the Research Associate provided additional details and scheduled a time to conduct the interview. Of the 70 eligible patients who were approached, 35 agreed to participate. Reasons for refusal included expressed lack of interest and lack of time. Of the 35 who agreed to participate 7 were removed from the sample leaving 28 participants. Of the seven removed, one was hospitalized, two were unable to understand questions due to cognitive impairment, one withdrew due to expressed lack of time, one had a scheduling conflict and another patient did not respond to the Research Associates to schedule an interview.

### Data collection

Before starting data collection the team received ethics approval from the Research Ethics Board of Sunnybrook Health Sciences Centre on Jan 23, 2012. One-on-one semi-structured interviews were then conducted with each of the patients, their family caregivers, and family physicians. Two of the co-authors (AG and GN) and Research Associates for the study, conducted the interviews. AG and GN are PhD and Master Candidates respectively in a Health Services Research program. They received training in qualitative methodology and had previous experience conducting interviews. They were mentored by the lead author (KK) who trained with the Health Experience Research Group (Oxford UK), a group of established qualitative researchers who collect and publish personal illness narratives. Interviews were conducted in English. Most interviews took place at the Family Health Team office in a private, designated space and a few were conducted in the patient’s home upon their request. Only two of the interviews required more than one visit to complete. All patient and caregiver interviews typically took one hour to 1.5 hours to complete. The physician interview guide was much shorter and took up to 30 minutes to complete. Only the interviewer and interviewee were present during the interview process. All interviews were conducted separately to ensure confidentiality of responses. The researchers had no previous relationship with the participants.

The Research Associate read from a script prior to asking the interview questions. In the script, the interviewer introduced herself as a Research Associate and described the intent of the study. The Research Associate assured the participant that the information collected was confidential and that they could stop the interview at any time without penalty and that it would not affect their future care. The participant was also reminded that the interview would be tape recorded. The Research Associates took notes during and after the interviews, which served as additional information if questions arose during thematic coding of the transcripts. Transcripts were not returned to participants for comment.

### Analysis

Both quantitative and qualitative descriptive analyses were conducted. Frequency counts and measures of central tendency were conducted to analyze patient and caregiver characteristics using SPSS version 17. Interviews were transcribed verbatim and thematically analyzed using computer assisted data analysis software (NVivo9). Qualitative data specific to patient goals of care were selected from the transcripts and thematically coded by a member of the research team (AG). This was achieved by selecting out the open ended responses to the patient survey question: “*Do you have care goals*?” *In other words*, *what would you say are your most important goals for*: *doing the things you want to do*, *staying in the best health you can attain*, *and living the life that you believe you can live*?” These responses were analyzed and compared to the open ended responses given by the caregiver and family physician on goals of care for the patient. Themes were derived inductively from the data and not identified in advance. Data analysis occurred simultaneously with data collection until saturation of themes occurred. Data saturation was determined when themes became repetitive within each of the patient, caregiver and physician groups. Themes started to become repetitive after the first 14 interviews.

To verify the themes identified by AG, two other members of the research team (KK and GN) read through the responses pulled from the transcripts, modified and aggregated the codes. Three researchers (AG, KK and GN) met on several occasions to review and discuss the themes until consensus was reached on the number and naming of themes. To discern the alignment of themes by each of the triads (i.e., patient, their caregiver and family physician) a similar process was undertaken whereby the researchers independently reviewed the responses across triads, assessed the extent of alignment and then met to discuss findings until consensus was reached. Since the question on goals was open-ended, participants tended to describe multiple goals, therefore it was possible for participants to demonstrate alignment on some goals and misalignment on other goals. Alignment was defined as concurrence on at least one goal.

## Results

As indicated in Table 1, patients had a mean age of 82.3 years; over half were male (56%); the majority were married (67%); Caucasian (96%); English speaking (89%); had more than high school education (70%), had sufficient financial resources (85%) and one-third (30%) lived alone. Patients had an average of 4.61 health conditions (*SD* = 2.43). As indicated in Table 2, most of the caregivers were female (82%); had an average age of 70.5 years; just over half were spouses of the patients (61%), one third were adult children (32%), one was a sibling (3.5%) and the other was a friend providing ongoing care (3.5%). The patients were linked to one of four physicians, three of whom were in practice for at least 10 years (Physicians A, B and D) with one physician (Physician C) who was newer to practice (1 year). No other demographic data was collected from the physicians. Table 3 outlines key characteristics of each of the 28 patient-caregiver-physician triads.

**Table 1:** Patient demographic data

**Demographic**		**Patient (n = 27)**
Age*		82.3 years (± 7.7 years)
Gender*		
	Female	13 (44%)
	Male	15 (56%)
**Marital Status**		
	Married	18 (67%)
	Other	9 (33%)
**Education**		
	High School Diploma or less	8 (30%)
	Greater than high school	19 (70%)
**Ethnicity**		
	Caucasian	26 (96%)
	Other	1 (4%)
**Language Spoken**		
	English	24 (89%)
	Other	3 (11%)
**Live Alone**		
	No	19 (70%)
**Type of Home**		
	Single/Family home	19 (70%)
	Apartment	4 (15%)
	Retirement Home	2 (7%)
	Other	2 (7%)
**Can Support Self Financially**		
	Yes	23 (85%)

*n = 28 for age and gender demographics.

**Table 2:** Caregiver demographic data

**Demographic**		**Caregiver (n = 28)**
**Age**		70.5 (± 11.3 years)
**Gender**		
	Female	23 (82%)
	Male	5 (18%)
**Relationship to Patient**		
	Spouse	17 (61%)
	Child	9 (32%)
	Sibling	1 (3.5%)
	Friend	1 (3.5%)

**Table 3:** **Patent**-**caregiver**-**physician triads**

**Patient**	**Age in years**	**Sex**	Number of reported health conditions*	**Caregiver relationship to patient**	**Caregiver age in years**	**Physician**
Patient 1	86	Male	1	Wife	77	A
Patient 2	79	Male	5	Wife	77	A
Patient 3	79	Female	4	Daughter	53	A
Patient 4	91	Female	4	Daughter	59	A
Patient 5	77	Male	3	Wife	74	A
Patient 6	85	Female	6	Daughter	56	B
Patient 7	77	Female	12	Husband	80	C
Patient 8	82	Male	7	Wife	77	C
Patient 9	70	Female	5	Sister	75	C
Patient 10	88	Female	2	Friend	80	C
Patient 11	83	Female	2	Daughter	50	A
Patient 12	68	Male	2	Wife	68	A
Patient 13	79	Male	4	Wife	58	D
Patient 14	85	Male	2	Wife	91	D
Patient 15	84	Male	3	Wife	84	D
Patient 16	75	Female	8	Husband	73	D
Patient 17	93	Female	5	Daughter	58	D
Patient 18	91	Female	4	Son	62	C
Patient 19	88	Male	1	Wife	88	A
Patient 20	80	Male	4	Wife	74	A
Patient 21	67	Male	4	Wife	65	A
Patient 22	73	Female	5	Husband	77	A
Patient 23	81	Female	6	Husband	71	A
Patient 24	83	Male	5	Wife	79	D
Patient 25	87	Male	8	Wife	87	D
Patient 26	94	Male	4	Daughter	61	B
Patient 27	96	Male	8	Daughter	63	D
Patient 28	84	Male	5	Daughter	58	B

* Although patients with more than one health condition were identified by physicians or administrators, two of the patients only reported one health problem during the interview.

Below, the key themes on goals derived from the patient, caregiver and family physician interviews are provided followed by the findings on alignment of goals across patient-caregiver-family physician triads.

### Patient goals

Patient goals fell into at least one of 4 themes: 1) health maintenance; 2) health improvement; and less commonly 3) behavior change; and 4) preparation for future needs.

Many patients were dealing with multiple chronic health problems for a lengthy period of time. In this context, many patients expressed a desire to maintain their current health and avoid decline. For example, a 77 year old married patient with mild cognitive impairment, hypertension, hyperlipidemia and obesity simply stated a desire to:

“*just to keep doing what I am doing basically*, *you know so stay in the same mode in mind that I am in right now*”. Patient 5

Similarly, an 84 year old married patient with Parkinson’s Disease, arthritis and other health conditions stated:

“*Well*, *I guess to keep me from falling victim to the problems of my disease any more quickly than is absolutely necessary*, *absolutely essential*”. Patient 15

Some patients were experiencing a particular symptom that was disruptive to their day to day living. In these cases their goal was to seek improvement or respite from the issue at hand. For example, a 79 year old married patient who was wheel-chair bound with an above knee amputation, diabetes, sleep apnea and other chronic conditions, pinpointed one specific symptom that he wanted to address (despite his myriad conditions):

“*If I have no pain*, *I am okay*. *I don*’*t care what I eat*, *who visits*, *who not visits*. *The main thing is if I don*’*t hurt*, *it*’*s fine*”. Patient 13

For others, seeking improvement was needed in order to resume their normal lives which included participating in recreational activities. An 88 year old widow who was preparing for her rectal surgery but also suffering from atrial fibrillation, and arthritis noted:

“*Well once it*’*s over*, *assuming the recovery is good*, *just getting back to everything I*’*m used to doing like the garden and the grass and the house and all the social activities I*’*m involved in*”. Patient 10

A few patients discussed a desire to change their behavior. A 77 year old married patient with cataracts, arthritis, heart problems, depression and other health issues talked about wanting “*to lose weight and to exercise*.” Patient 7. Another patient who was married and 75 years of age with diabetes, arthritis and glaucoma wanted to, “*be less of a burden*”. Patient 16

A couple of patients acknowledged that they reached a point where they could not longer remain at home on their own. Either home support or a transition to a long-term care facility was required. In these two cases, preparation for next steps was noted:

An 83 year old widowed patient talked about wanting to “*get rid of things*, *a lot of things*, *because* [*she was*] *moving into a seniors residence*…” Patient 11. The need for more support was not always a welcomed transition, particularly for a 96 year old widow with high blood pressure, chronic back pain and a rare bone disease who stated:

“*I*’*ve got to either get somebody in here to stay*, *to live with me*, *or go to a care facility*. *And I prefer to stay here and get somebody to come in*. *End of story*”. Patient 27

### Caregiver goals

Caregiver goals for the patient fell into 6 themes. The first three goals aligned with patient goals: 1) health maintenance, 2) health improvement/symptom management and 3) preparation for future needs with the added nuance of having the care recipient accept these added services. Other goals included 4) doing tasks for the patient; and in a few cases 5) keeping the patient safe and 6) helping them maintain dignity, particularly at the end of life.

The goal of health maintenance was required for care recipients to do their usual activities including maintaining a social network. A 77 year old spousal caregiver of an individual with several health conditions including stroke, COPD, neuropathy and bladder cancer stated:

“*he would love to be able to walk again*. *His mobility is something that*’*s very important to him*. *Our grandchildren*, *our family is very important*. *And because we have a summer cottage this is sort of a gathering point for the family*, *it*’*s important to him and to me too to be able to assemble there and do things*”. Caregiver of Patient 2

Other goals were more specific to symptom management. A 73 year old spousal caregiver for an individual with congenital blindness, depression, diabetes and osteoarthritis plainly stated:

“*well it would be good to get rid of the pain from the sciatica*”. Caregiver of Patient 16

Some caregivers expressed the goal of getting more care supports in place. For example, a 58 year old spousal caregiver for a 79 year old patient who was wheel-chair bound with an above knee amputation, diabetes, sleep apnea and other chronic conditions (introduced earlier in the paper) stated:

“*Well the most important for him is someone is there to look after him*, *someone is there that if he needs help with something that he knows that he is not alone and cannot do this for himself*”. Caregiver of Patient 13

In some cases, patients were resistant to such care so caregivers expressed a desire for patients to accept outside services. A 50 year old caregiver who was looking after her mother who was diagnosed with depression, compression fractures and heart failure shared her struggle:

“*trying to convince her that it*’*s a safer way that she can do things independently instead of she looks at it as something that*’*s showing people she*’*s an invalid*, *shall we say*.” Caregiver of Patient 11

With the intent of supporting the patient as much as possible, some caregivers expressed the desire to continue to do tasks for the patient. A 77 year old spousal caregiver of a patient with arthritis, hypertension, and cancer shared the following:

“*So my goal certainly is to support him to go to all of his appointments and to keep track of his health*, *and to feed him well*”. Caregiver of Patient 2

When some of the caregivers spoke about their desire to continue to care for the patient they also acknowledged their own levels of anxiety and stress in relation to their role as a caregiver. For example, a 74 year old spousal caregiver’s goal was “*just to stay sane*.” Caregiver of Patient 20

Among caregivers who were looking after patients with more serious impairments including dementia, safety and dignity were integral:

“*My goal is for him to be safe*. *Because he doesn*’*t walk*, *I*’*m concerned that if he tries to get out of bed or forgets that he can*’*t walk they he might have a fall*”. Caregiver for Patient 8

Lastly, a daughter who was caring for her 93 year old mother who was nearing the end of life noted:

“*But as she has said on many occasions*, *she would like to die with dignity*. *I think she realizes she*’*s sort of at the latter stages of her life now*”. Caregiver of Patient 17

### Family physician goals

Family physician goals fell into 4 themes, which aligned with both patients and caregivers: 1) to help maintain independence of the patient, 2) to heal, fix or improve symptoms when possible, 3) mobilize care for the patient and the caregiver and 4) to address safety issues. With these goals in mind, the physicians wanted to prepare the patient and caregiver unit for anticipated decline.

Many physicians wanted to help the patient maintain his or her independence. One physician stated:

“*My main goal of care would be to keep her as high functioning as possible at home*”. Physician of Patient 6

Many physicians also wanted to heal, fix or improve patient symptoms and ailments whenever possible. While the presence of multiple co-occurring conditions may limit the extent to which improvements are possible, in some cases, patients were experiencing a particular flare up or symptom that had to be addressed. In reference to a patient recovering from surgery, a physician stated:

“*I think it*’*s just to acutely get him through some humps*, *surgical humps*”. Physician of Patient 2

Aside from functional improvements or maintenance, family physicians acknowledged the importance of having the right supportive infrastructure in place. Mobilizing services for patients and (in some cases) caregivers, was important to maintain patients safely in their homes. As stated by one physician:

“*If she*’*s going to stay at home*, *she needs to have more resources at home*, *and she*’*s going to need to accept them*”. Physician of Patient 11

Some physicians were concerned for caregivers, particularly those who were aging themselves and appeared to be at risk of stress. One physician noted:

“*And so one of my goals would be to have her* [*patient*] *healthy enough so that she*’*s not killing her daughter because her daughter is stressed helping her manage*”. Physician of Patient 6

When patients were declining physically or cognitively, physicians tended to emphasize preparation for worsening health as a key goal:

“*So goals of care*, *I also would say the big thing would be to sort of prepare him as his dementia worsens*, *for both him and his wife*, *who*’*s not my patient but I*’*m conscious that it has a huge impact on her*”. Physician of Patient 1

### Alignment of goals

At an aggregate level there was some consistency across patient, caregiver and family physician responses in the identified goals, particularly around symptom alleviation and maintaining health status. A couple of patients made reference to the need for more services, but this was more commonly expressed among caregivers and physicians. Table 4 outlines the extent to which themes aligned for each of the three groups.

**Table 4:** **Goals Reported at the aggregate level among patients**, **caregivers and physicians**

	**Health maintenance**	**Health improvement/symptom management**	**Behaviour change**	**Preparation for future needs/more or different services**	**Doing tasks for the patient**	**Safety and dignity**
Patients	X	X	X^a^	X^a^		
Caregivers	X	X		X	X	X^b^
Physicians	X	X		X^c^		X^b^

^a^ Expressed by a minority of patients.

^b^ For patients with unstable or declining health.

^c^ Physicians also recommended care for the caregiver.

Alignment was less evident when comparing responses across triads. Themes were compared across 27 of the 28 triads because one patient interview was incomplete. In a minority of the triads, there appeared to be alignment in at least one goal across all three parties. When alignment was found, patients tended to have stable health conditions or a very specific ailment or “flare-up” that required attention. For some patients, recognition of the need to transition to a new care setting like a long-term care home was acknowledged and all parties agreed that this was the best option. In most of these cases, there was also acknowledgement that when the time arose, some preparation for inevitable decline would be required.

In the following excerpt a 68 year old married patient with Crohn’s disease, his 68 year old spousal primary caregiver and family physician show congruence in their care goals for the patient:

### Patient 12

“*Well probably to get the Crohn*’*s more manageable would be*…*and the other things tend to fall into place*…”

### Caregiver for Patient 12

“…*just to manage his disease*. *I want to make sure that his weight is kept up*. *His doctors are pretty good about looking after him*…”

### Family physician for Patient 12

“*I think he has a lot of exacerbations of his Crohn*’*s disease*. *So I think the goals of care*, *he*’*s trying new treatment right now hoping to keep him so that he*’*s not in hospital all the time with complications related to his Crohn*’*s*”.

In this example each party makes reference to the patient’s disease. While the caregiver and family physician provided more context to the health issue (e.g., weight control, new treatment, etc.), the main goal across all three individuals is to manage the symptoms of the patient’s Crohn’s disease. In cases such as Crohn’s, a condition that tends to be clinically dominant, it may trump or eclipse the care of other conditions; which may be the case here.

Conversely, lack of alignment tended to occur when patients had unstable functional or cognitive health, when safety threats were noted, need for services was expressed but not all parties were on board (including potential transfer to facility based long-term care) or when caregiver burnout was detected. The following excerpt provides an example of an 85 year old patient with dementia, his 91 year old spousal caregiver and the family physician. Differences in care goals for the patient are expressed:

### Patient 14

“*Staying alive*…*to stay positive and upbeat*…”

### Caregiver for Patient 14

“…*help with the memory loss*, *improving memory*, *he still enjoys social contacts*…”

### Family physician for Patient 14

“*So safety is a big concern for him*. *He lives with his elderly wife who*’*s the primary caregiver*. *So she*’*s at huge caregiver burnout risk there*. *And most recently*, *he*’*s always had sort of outbursts of anger where he would*, *you know*, *hit things or throw things but not directed at her*. *But more recently she expressed some concern that*, *you know*, *he may actual direct it at her*; *so I guess my goal of care is to try to come up with a good long term care plan*”.

In this example, the patient, who has cognitive impairment, provides a straight forward response about simply “staying alive”. The caregiver is aware of memory loss and expresses a desire to improve it and maintain his social contacts; while the physician acknowledges a growing safety threat for both the patient and caregiver and the need for a long term plan. Arguably, all of these aspects are important to consider for the patient, but the differences may warrant a discussion between these parties in order to build a plan to move forward. We elaborate on this and other insights in our discussion below.

## Discussion

Goal setting in clinical care is recommended as a means to support individuals with multi-morbidities and their caregivers in managing their conditions. In this paper, the types of goals that were important for older persons with multi-morbidities were explored from the perspectives of patients, their caregivers and physicians. Comparisons of goals were made across each patient, caregiver and physician triad to determine alignment. As a first step, patient, caregiver and physician goals were examined at the aggregate level; in other words, all of the patient data on goals of care was examined thematically, followed by all of the corresponding caregiver data and then all of the physician data. Maintaining functional independence for as long as possible was a common goal expressed by all three parties. In addition, managing or fixing specific symptoms or functional challenges that were hindering activities of daily living and social outings were commonly expressed, especially among caregivers and physicians. The importance of maintaining independence align with findings by Fried et al. [14] and Bogardus et al. 2001 [16] where autonomy and day to day functioning respectively were stated as the most important care goals for older persons and/or their caregivers.

Differences were also found at the aggregate level. While patients tended to focus on their own symptoms and functions in relation to their goals, caregivers tended to focus on creating the needed context to foster patient independence. The needed context included home adaptations, the maintenance of social connections (e.g., social outings), and formal homecare support. Some caregivers, particularly those who were feeling stressed, noted that their own personal perseverance was necessary to adequately support the patient. For patients in the later stages of illness, maintaining safety and dignity were added to the caregiver’s goals list. Physician goals for the patient included safety and future planning (mobilizing the right services in the appropriate setting) with acknowledgement of the needs of the caregiver as well (particularly if caregiver stress was detected). These goals occurred alongside the goals of alleviating symptoms at a clinical level for the patient to optimize health.

While similarities and differences were noted at the aggregate level a different picture emerged when examining the alignment of care goals across each of the patient, caregiver and family physician triads. Alignment of at least one goal across all three parties was shown in a minority of cases only. Low agreement on care goals have been found in previous research. Heisler et al. [17] examined the alignment of diabetes treatment goals among 27 diabetic patients and their physicians and found low agreement on top treatment goals. Bogardus et al. [16] surveyed family physicians and caregivers of 200 geriatric outpatients and found that overall alignment of treatment goals was poor, but 79% of the dyads aligned on at least one goal. Our study was different in that we were examining the alignment with patients as well, which is perhaps why alignment on at least one goal in our study was considerably lower.

In our study, when alignment on at least one goal did occur it tended to be when patient conditions were stable or when a very specific symptom or acute exacerbation was evident and had to be addressed. Such specific issues presumably surfaced in a more obvious fashion during interactions with the patient. The tendency for parties to align on one goal but not others was likely a product of the open ended nature of the interview question. Unlike previous research that used closed ended response categories to capture and compare goals [14,16,17], our open-ended approach allowed patients, their caregivers and family physicians to elaborate on their answers and speak about multiple goals. For example, while all parties in a triad may agree on maintenance of physical functioning, it was typically the caregiver and/or physician who would emphasize the importance of *also* preparing for future decline.

Alignment less likely occurred when patients had significant illness complexity, such as unstable, fluctuating health problems or cognitive decline which posed immediate or anticipated threats to the safety of the patient or caregiver. In these cases enhanced services (in the home or in a long-term care facility) were recommended to support the person and/or the caregiver. In other words, when the patient became less stable and future steps were uncertain, divergence in care goals tended to occur.

A key question arises from these findings: “Do countervailing goals and strategies represent threats to patient self-management or are they necessary differences that align with the roles and experiences of each member of the care team?” For example, in the case of the patient with dementia detailed in the findings (Patient 14), a clear lack of goal alignment was noted. It appeared that the patient was unaware of the full extent of his decline and inability to self-manage. The caregiver, who presumably had greater insight into the capabilities of the patient acknowledged the patient’s memory loss but wanted to maintain his social contacts. The caregiver appeared to be protecting the capability of the patient with less emphasis on the potential severity of the situation. The physician, mostly concerned about safety and caregiver burnout, recommended a long-term care plan. These necessary differences in goals represented a security-liberty trade-off. While the patient and caregiver are focusing on securing the patient’s ability to age in the community, the physician acknowledges concern over the future safety of the caregiver and patient. Value trade-offs such as this one represents a potential ethical issue that may be faced in practice. We lack a formal account of these issues even though they represent a key component in caring for this population.

When individuals have multiple chronic health issues with the added complexity of fluctuating symptoms and declining health, convergence in goals between them, their caregivers and physicians may not be possible or realistic. Perhaps it is more important to create the space in clinical practice for a conversation to take place on the identification and prioritization of goals between physicians, their patients and caregivers. For example, Fried et al. [14] recommend health outcome prioritization between physicians and patients as a means to target care plans accordingly for individuals with multi-morbidities. Future observational studies of patient-physician consultations may be a useful way to examine the extent to which goal-setting characterizes the discussion and how value trade-offs and prioritization of goals is addressed, if at all. The consideration of patient, caregiver and family physician perspectives during clinical consultations will surely elucidate different strategies and perspectives that ultimately aim to optimize the care and safety of the patient and caregiver.

Resources to support research in the development and evaluation of techniques are needed to foster goal explication and alignment. Innovative approaches to primary care delivery for patients with multi-morbidities such as the Inter-professional Model of Aging and Complex Treatments (IMPACT) [22], and other team based and multi-disciplinary approaches to treatment and care may be appropriate venues for implementing and testing goal setting strategies for individuals with complex health issues that move beyond the traditional patient-physician consultation.

## Conclusions

The data suggest that goal divergence tends to occur when patients are less medically stable. While goal divergence may be expected due to the different roles and responsibilities of each of the players involved, these perspectives should be illuminated when building care plans. Further research is required to observe the extent to which goal setting occurs in family practice as well as how it can be embedded as a standard of practice.

### Limitations

The study took place in one urban-based family medicine clinic situated in a particularly affluent neighborhood. The sample in this study was mostly Caucasian, English speaking, well educated and financially secure, which may limit the transferability of our findings to other populations that do not match this demographic profile. Further research is required in other primary care settings that serve a more culturally diverse patient population to discern if different goals emerge as a product of culture, socio-economic status and other related factors. Nonetheless, the findings reported here highlight new knowledge about important ways that physicians, patients, and caregivers differ in their respective goals regarding patients’ health that have implications for clinical practice, policy and research.

## Competing interests

The authors declare that they have no competing interests.

## Author’s contributions

KK contributed to the study design and interpretation of the data and took the lead on drafting and revising the manuscript. AG and GN took the lead on data collection, data analysis and interpretation and contributed to manuscript revisions. RU, LJ and WW contributed to the study design and revisions of the manuscript. All of the authors read and approved the final version of the manuscript.

## Pre-publication history

The pre-publication history for this paper can be accessed here:

http://www.biomedcentral.com/1471-2296/14/133/prepub
